# Microvascular Inflammation of the Renal Allograft: A Reappraisal of the Underlying Mechanisms

**DOI:** 10.3389/fimmu.2022.864730

**Published:** 2022-03-22

**Authors:** Emilie Lebraud, Maëva Eloudzeri, Marion Rabant, Baptiste Lamarthée, Dany Anglicheau

**Affiliations:** ^1^ Necker-Enfants Malades Institute, Inserm U1151, Université de Paris, Department of Nephrology and Kidney Transplantation, Necker Hospital, AP-HP, Paris, France; ^2^ Department of Renal Pathology, Necker Hospital, AP-HP, Paris, France; ^3^ Université Bourgogne Franche-Comté, EFS BFC, Inserm UMR1098, RIGHT Interactions Greffon-Hôte-Tumeur/Ingénierie Cellulaire et Génique, Dijon, France

**Keywords:** antibody-mediated rejection (ABMR), microvascular inflammation (MVI), anti-HLA donor-specific antibodies (HLA-DSA), non-HLA antibodies, kidney transplantation

## Abstract

Antibody-mediated rejection (ABMR) is associated with poor transplant outcomes and was identified as a leading cause of graft failure after kidney transplantation. Although the hallmark histological features of ABMR (ABMRh), i.e., microvascular inflammation (MVI), usually correlate with the presence of anti-human leukocyte antigen donor-specific antibodies (HLA-DSAs), it is increasingly recognized that kidney transplant recipients can develop ABMRh in the absence of HLA-DSAs. In fact, 40-60% of patients with overt MVI have no circulating HLA-DSAs, suggesting that other mechanisms could be involved. In this review, we provide an update on the current understanding of the different pathogenic processes underpinning MVI. These processes include both antibody-independent and antibody-dependent mechanisms of endothelial injury and ensuing MVI. Specific emphasis is placed on non-HLA antibodies, for which we discuss the ontogeny, putative targets, and mechanisms underlying endothelial toxicity in connection with their clinical impact. A better understanding of these emerging mechanisms of allograft injury and all the effector cells involved in these processes may provide important insights that pave the way for innovative diagnostic tools and highly tailored therapeutic strategies.

## Introduction

In kidney transplantation, antibody-mediated rejection (ABMR) remains one of the major causes of graft loss (1). Successive Banff conferences, the most recent of which was held in 2019, have established an international classification of rejections, dichotomizing acute rejection mediated by T cells (TCMR) and ABMR (humoral rejection). Currently, 3 criteria must be met to identify ABMR: microvascular injury (MVI), evidence of current/recent interaction between an antibody and the vascular endothelium, and serological evidence of donor-specific antibodies (DSAs), which can be specific for human leucocyte antigens (HLAs) or non-HLA antigens (2). MVI illustrates the leukocyte margination reaction in the glomeruli (glomerulitis, Banff score g) and peritubular capillaries (peritubular capillaritis, Banff score ptc). The Banff classification introduced an MVI score (g+ptc) depending on the context. In the absence of C4d staining, the MVI score must be ≥ 2. In the case of concurrent TCMR or borderline TCMR, ptc and g must be present to establish a diagnosis of ABMR (3). While the prototypic presentation of ABMR includes both MVI and circulating anti-HLA DSAs (HLA-DSAs), the observation of histological features of ABMR in the absence of detectable HLA-DSAs remains puzzling to the transplant physician, and in 2019, Senev and colleagues introduced the term ABMRh to characterize patients with histological features of ABMR in the absence of HLA-DSAs (4). Despite the development of increasingly specific and sensitive assays, many studies have shown that circulating HLA-DSAs are often absent in recipients who nevertheless present with ABMRh (4–10). It is now assumed that 40-60% of patients with ABMR do not have circulating HLA-DSAs (4, 6, 7). In addition, the clinical phenotype could be different, since patients without HLA-DSAs are associated with more transient histological lesions and improved overall graft survival compared to HLA-DSA-positive patients (4, 11). However, several reports also demonstrated that allograft biopsies with MVI in the absence of HLA-DSA might exhibit unusual frequency of vasculitis lesions, more severe vasculitis scores or even thrombotic microangiopathy or interstitial hemorrhages, suggesting a dramatic involvement of the vascular wall (8, 12–14). This observation suggests that non-HLA-mediated pathogenic mechanisms targeting graft endothelial cells might exhibit particular phenotypes of vascular rejections with severe endothelial/vascular injury.

Taken together, these observations highlight the increasing need for transplant physicians to identify the underlying mechanisms of graft injury to ultimately improve ABMR treatment and long-term graft outcome. In this review, we aim to present basic knowledge and recent findings on the mechanisms, effector cells and molecules involved in ABMR-induced MVI. How anti-HLA and non-HLA-specific antibodies trigger vascular injury, which mechanisms are required, and consequently how these mechanisms influence kidney allograft rejection are of great interest for better addressing biopsies presenting criteria inconsistent with the Banff classification system.

## HLA-DSA-Dependent MVI

### HLA-DSAs and ABMR

**Figure 1:**
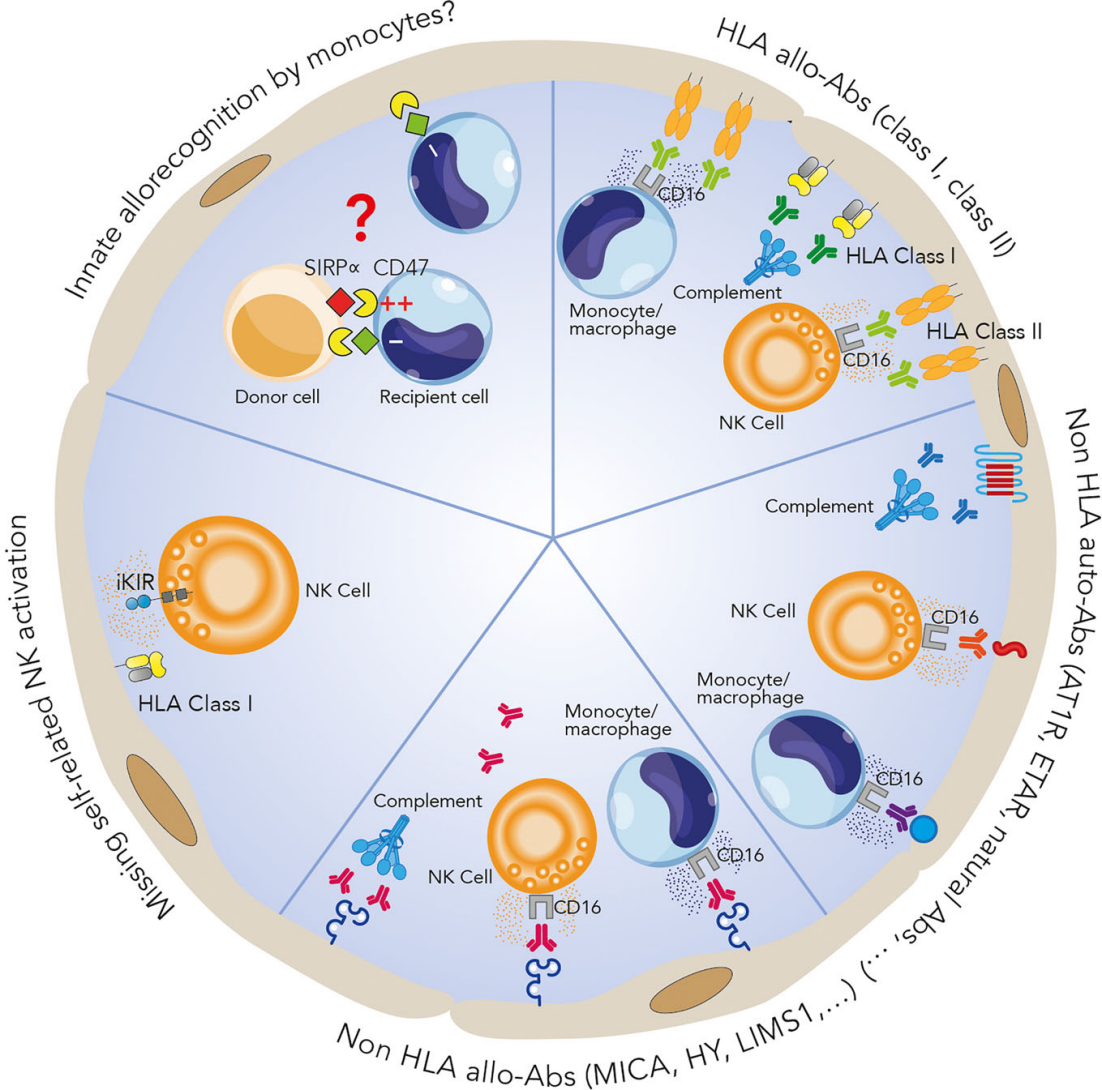
Mechanisms potentially involved in microvascular inflammation. HLA-DSAs can cause microvascular damage through activation of the complement system and recruitment of inflammatory cells such as NK cells and monocytes/macrophages *via* their crystallizable fragment (Fc) receptors, inducing antibody-dependent cell cytotoxicity. Many non-HLA allo- and autoantibodies have also been identified as players in allograft rejection. The exact mechanisms involved are still unclear but could be similar to those of HLA-DSA-associated processes. Recent studies have identified antibody-independent mechanisms involving the key role of innate immune cells distinguishing between self and non-self, leading to an alloimmune response: NK cell activation can render these cells apt to attack endothelial cells *via* a missing self-mechanism, while a SIRPα/CD47 mismatch between the donor and recipient can lead to monocyte allorecognition and monocyte-driven graft injury.

DSAs are defined as antibodies that recognize a “non-self” peptide in the recipient graft. In transplantation, the current dogma is that alloantigens expressed by the graft are recognized by DSAs directed against class I and II HLA antigens (**Figure 1**). HLA- can easily be fixed to major histocompatibility complex (MHC) alloantigens expressed on the surface of endothelial cells in the graft. The renal endothelium is particularly prone to injury in kidney transplant recipients, as endothelial cells from the transplant are directly exposed to the recipient’s immune system. The presence of DSAs that react with the mismatched donor’s HLA type causes MVI, leading to ABMR and kidney graft failure (15, 16).

As mentioned above, the detection of HLA-DSAs is essential for diagnosing ABMR. The first technique for the detection of HLA-DSAs in recipients prior to kidney transplantation was introduced in the 1960s with complement-dependent cytotoxicity (CDC) crossmatch (17). Since then, progressive improvements in sensitivity have culminated in the HLA antigen-coated fluorescence bead assay based on a Luminex platform (Luminex single bead or SAB) (18). In the latter, an extremely sensitive assay, the mean fluorescence intensity (MFI) is used as a measure of the degree of saturation of total antigens present on the beads bound by antibodies. This MFI is often used as a surrogate marker for antibody titers. However, one current challenge is the lack of consensus in defining an optimum MFI cutoff to discriminate positive and negative values (18–22). In addition, the MFI values of this cutoff depend on the Luminex instrument and reagent kits, which are laboratory specific, making ABMR diagnoses with circulating HLA-DSAs difficult to compare among centers. In addition, accumulating evidence suggests that MFI should not be considered a surrogate of antibody titer, and several factors influencing the MFI value have been identified. For example, interference mediated by the activated complement components can cause false negative or reduced MFI levels by competitively displacing the detection antibodies (phenomenon also called prozone effect) (23, 24). Serum dilution or serum treatment with reagents such as ethylene-diamine-tetraacetic acid (EDTA) (25) can prevent this effect but laboratory practices are once again not shared by all the centers. Finally, substantiating these analytical issues, several studies have found an increased risk of graft loss with high titers of DSAs (26, 27), while other did not found any clear association between MFI values and graft survival (28–30). Finally, the causal relationship between circulating HLA-DSAs and ABMRh has long been a matter of debate (31), but accumulating evidence strongly supports the causality of HLA-DSAs in ABMR (32).

Transplant recipients with pre-existing HLA-DSAs are at a higher risk of ABMR than those without circulating DSAs (33–36), but the posttransplant evolution of preformed HLA-DSAs seems to be associated with long-term graft outcome. In a cohort of 924 patients, Senev et al. showed that 52% of the patients with pretransplant HLA-DSAs had resolution of these pre-existing antibodies within the first 3 months posttransplant. The persistence of pretransplant HLA-DSAs had a negative impact on graft survival (36). The development of *de novo* HLA-DSAs occurs in 10-40% of solid organ transplant recipients (37), and the presence of these antibodies is strongly associated with allograft injury and reduced graft survival (38–41). In a large study of 771 kidney biopsy specimens, Aubert et al. showed that the development of *de novo* HLA-DSAs was associated with an increased incidence of chronic allograft injury. Graft biopsies of patients with *de novo* HLA-DSAs were characterized by increased expression of IFNγ-inducible, NK cell-related, and T cell-related transcripts compared to those of patients with ABMR with pre-existing HLA-DSAs (42). These findings suggest differences in the mechanisms that drive alloimmune injury associated with pre-existing or *de novo* HLA-DSAs. However, the strategies to manage *de novo* HLA-DSAs are identical to those for managing pretransplant HLA-DSAs.

### Antibody Producers: Memory B Cells and T Follicular Helper (TFH) Cells

Circulating antibodies are produced constitutively by long-lived plasma cells residing in the bone marrow and by circulating specific memory B cells (mBCs) that rapidly differentiate into antibody-secreting cells upon antigen reexposure (43). B cell activation occurs in the secondary lymphoid organs, such as the spleen and lymph nodes. Two signals are necessary for this activation: recognition of the cognate antigen through the B-cell receptor and engagement of CD40/CD40L with cognate TFH cells (44). mBCs can be generated by T cell-dependent activation through both the extrafollicular response and the germinal center reaction (45). Much of the quantification of mBCs in transplant patients has focused on the total mBC population in the peripheral blood, irrespective of mBC specificity. Although phenotypic identification of mBCs is being increasingly refined, a significant limitation of this approach is that it fails to assess the frequency of donor‐reactive B cells, which are the clinically relevant population. Thus, a number of approaches have been developed to address this limitation; these strategies were reviewed by Chong et al. (46).

Concerning the anti-HLA humoral response, Luque et al. developed an HLA B cell enzyme-linked immunospot (ELISpot) assay to monitor circulating donor-reactive mBCs after transplantation. Markedly, 21 of 29 (72.4%) HLA-DSA-negative patients with chronic ABMR had circulating donor-reactive mBCs (6, 47). This finding assumes that mBCs can be involved in ABMRh, even in the absence of circulating HLA-DSAs at the time of biopsy, and that measuring mBCs and HLA-DSAs may allow better discrimination of patients with ABMRh. Moreover, in a longitudinal cohort of 90 nonsensitized patients with 6- and 24-month protocol biopsies, 1 of 5 patients with subclinical ABMR were mBC positive but HLA-DSA negative at 6 months, as were 2 of 12 at 24 months (47). This study suggests that independent of HLA-DSAs, interrogation of mBCs may improve risk stratification.

As mentioned above, TFH cells are crucial in promoting antibody production by B cells (48) and could also be involved in allograft rejection. In the context of kidney transplantation, recent reports have shown an association between circulating TFH (cTFH) cell profiles and humoral alloreactivity (49, 50). Notably, cTFH cells, i.e., CD4^+^CXCR5^+^ TFH-like cells, can predict HLA-DSA formation after transplantation. Indeed, La Muraglia et al. observed expansion and differentiation of donor-reactive CD4^+^CXCR5^+^ cTFH cells following kidney transplantation using murine transplant models. Expansion and differentiation temporally correlated with germinal center alloreactivity and preceded the generation of DSAs (51). Thus, monitoring cTFH cells, more specifically, the activated CXCR5+PD1+ICOS+ cTFH cell subset linked to antibody production, in transplant patients may predict the development of ABMR (52–55). However, studies addressing the potential role of cTFH cells in non-HLA antibody production are still required.

## Non-HLA Antibody-Dependent MVI

**Figure 2:**
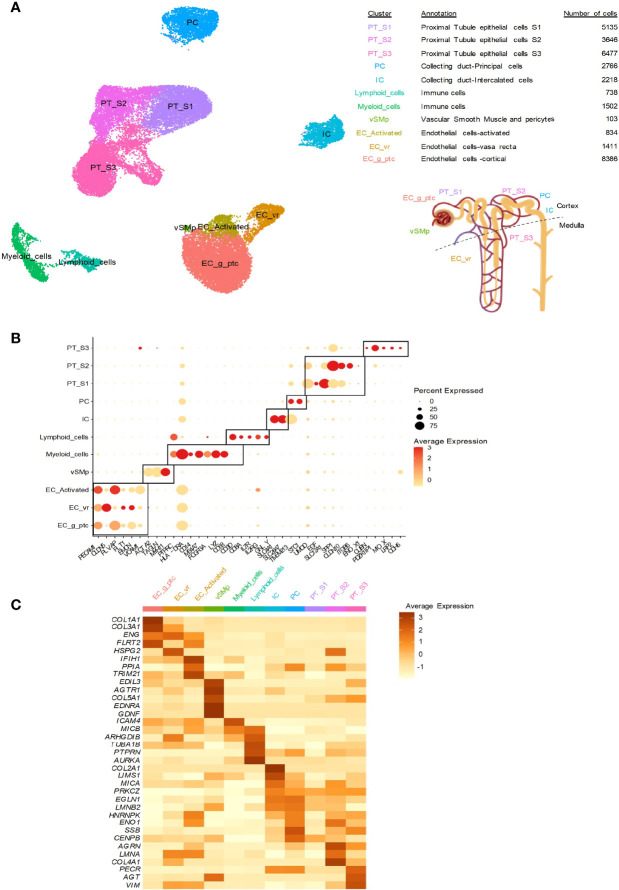
Kidney distribution of non-HLA antigens at the transcriptional level. Previously published human single-cell data from two ABMR biopsies and four healthy references corresponding to transplant surveillance biopsies were used. The associated raw counts or matrices were downloaded from the Gene Expression Omnibus (GEO; GSE145927, https://www.ncbi.nlm.nih.gov/geo) and Kidney Precision Medicine Project (https://atlas.kpmp.org/repository). The analysis was performed as previously published (67, 68). **(A)** UMAP dimensionality reduction for the different cell type clusters identified by scRNA-seq analysis. The number of cells in each cluster and the location of the cell population in the kidneys are provided. **(B)** Dot plot showing selected cell marker gene expression (dot color scale) and the within-cluster detection rate (dot size). **(C)** Heatmap showing the average expression of the indicated non-HLA antigens across the cell clusters.

As mentioned above, ABMRh without detectable circulating HLA-DSAs suggests the involvement of antibodies directed against non-HLA antigens called minor histocompatibility antigens. Despite the fact that the presence of these antibodies is now increasingly accepted (56–61), the real issue is whether these antibodies can cause endothelial damage on their own or whether they are biomarkers of previous/ongoing injury (62). Two categories of non-HLA antibodies have been identified. Alloantibodies are directed against polymorphic antigens, which differ between the donor and the recipient, and autoantibodies are antibodies that recognize self-antigens (**Figure 1**). Many antigens have been identified, including angiotensin II type 1 receptor (AT1R), endothelin receptor type A (ETAR), perlecan (LG3), intercellular adhesion molecule 4 (ICAM4), endoglin, EGF-like repeats and discoidin I-like domains 3 (EDIL3), and protein kinase Cζ. Excellent reviews detailing the current knowledge on non-HLA antibodies in transplantation have already been published (60, 63–66). Here, to investigate the distribution of non-HLA antigen expression in the kidneys, we performed single-cell analysis of previously published human single-cell data (**Figures 2A, B** **)** from two ABMR biopsies and four healthy references corresponding to transplant surveillance biopsies as previously described (67). We focused on the main studied antibodies and discuss new large-scale studies in the field of non-HLA antibodies.

### MHC Class I-Related Antibodies

Major histocompatibility class I-related chain A (MICA) and chain B (MICB) are alloantibodies among the most polymorphic non-HLA antigens, with more than 200 MICA and MICB alleles. These antigens, especially MICA, have been extensively studied in kidney transplantation (**Figure 1**). *MICA* expression was previously found on endothelial cells, epithelial cells, fibroblasts, dendritic cells, monocytes and keratinocytes. In the kidneys, we observed *MICA* expression mainly in intercalated cells (ICs), peritubular (PT) cells and activated endothelial cells. In contrast, *MICB* was mainly expressed in immune cells and the endothelium but not in the epithelium (**Figure 2C**). MICA acts as a ligand for NK cell group 2 (NKG-2D), activating receptors on natural killer (NK) cells, γδ T cells and CD8+ αβ T cells, thereby mediating innate cellular cytotoxicity (69). MICA-specific antibodies can also activate complement, resulting in antibody-dependent cellular cytotoxicity (ADCC), which causes injury through adaptive immunity. They can be a result of prior transplantation, pregnancy, or transfusions, as for HLA-DSAs (70).

Whether MICA-specific antibodies are pathogenic in kidney transplantation is still controversial, and evidence showing that *MICA* allele mismatching between the donor and recipient affects ABMRh and long-term graft outcome through the development of donor-specific MICA antibodies still needs to be produced. Filippone et al. recently described the current knowledge from studies considering the effects of pre- and posttransplantation MICA-specific antibodies on kidney transplant outcome (66).

### G-Protein-Coupled Receptors

AT1R is probably the most studied antigen that acts as a target of non-HLA antibodies. AT1R is a G-protein-coupled receptor encoded by *AGTR1* that binds to angiotensin II, leading to hypertension, vasoconstriction and vascular smooth muscle migration and proliferation (71). At the single-cell transcriptomic level, we found that *AGTR1* was mainly expressed in vascular smooth muscle and pericytes (vSMp), as expected. In 2005, Dragun et al. first reported elevated levels of AT1R-specific antibodies in transplant recipients with severe steroid-resistant vascular rejection and malignant hypertension in the absence of HLA-DSAs (72). In that study, among the recipients without DSAs, 16 tested positive for anti-AT1R antibodies and presented vascular injury and hypertension. Notably, graft biopsy samples from AT1R-positive patients did not show evidence of complement deposition. Several studies have since confirmed these results and showed correlations between anti-AT1R antibodies and an increased incidence of ABMRh and decreased graft survival. The reader is referred to a recent review by Zhang and Reed for more details about these studies (60).

These antibodies may be present at the time of transplantation or develop *de novo* after transplantation. In 2013, Taniguchi et al. studied the presence of pre- and posttransplantation AT1R-specific antibodies in 351 kidney transplant recipients using a cutoff value of 15 U/ml. The patients who were positive for anti-AT1R antibodies before transplantation (9.9%) but became negative after transplantation did not show an impact on graft survival. However, 48% of the patients with anti-AT1R antibodies before and after transplantation and 64% of the patients who developed *de novo* anti-AT1R antibodies suffered graft loss (73). Interestingly, the patients who had both anti-AT1R antibodies and HLA-DSAs exhibited poorer graft survival than those with HLA-DSAs alone, suggesting a synergistic effect for these two types of antibodies. Nevertheless, the clinical significance of these antibodies is still controversial. A possible explanation could be found in the interpretation of data with an appropriate cutoff for positivity. Indeed, most studies use a cutoff of 10 U/ml or 17 U/ml, but there is no consensus among centers (74).

More recently, several teams have investigated the potential role of anti-AT1R antibodies in the absence of HLA-DSAs. In 2018, Min et al. studied 359 kidney transplant recipients and found that MVI was the most common histological feature of allograft failure in patients with pretransplant anti-AT1R antibodies. Among these patients, six were MVI positive, HLA-DSA negative, and C4d negative but developed allograft failure (75). One year later, Lefaucheur et al. measured HLA-DSAs and anti-AT1R antibodies in 1845 kidney transplant recipients at the time of rejection and at 1 year posttransplant. Among 77 HLA-DSA-negative ABMRh patients, 51 (66%) had anti-AT1R antibodies. These patients had a higher prevalence of hypertension, more vascular rejection with arterial inflammation and higher levels of endothelium-associated transcripts than those with HLA-DSAs (12). Once again, they had no C4d deposition, suggesting that complement-independent mechanisms contributed to graft injury.

ETAR is another G-protein-coupled receptor identified as an endothelial antigen. It is encoded by the gene *EDNRA*, which is also mainly expressed in vSMp. The phenotype of anti-ETAR antibody-related transplant injury has not yet been precisely defined. However, a prospective study evaluated the presence of anti-ETAR antibodies in 116 consecutive kidney transplant recipients through pre- and posttransplant screening. They found ETAR-specific antibodies in 55 (47%) of the recipients before transplantation. The presence of anti-ETAR antibodies was associated with worse renal transplant function during the first 12 months after transplantation (76).

In transplant contexts, AT1R- and ETAR-specific antibodies could play a role in liver, heart and lung transplantation (61). AT1R and ETAR were also found in nontransplant patients and associated with cardiovascular disease, preeclampsia, cancer and autoimmune disease (77). They are therefore considered autoantibodies, recognizing self-antigens, rather than alloantibodies (**Figure 1**).

### Perlecan/LG3

Perlecan is a proteoglycan found within the vascular basement membrane and thus not a surface endothelial antigen *per se*. It is encoded by the *HSPG2* gene, which is mainly expressed by vasa recta endothelial cells (ECs-vr) and PT cells (**Figure 2C**). The third laminin-like globular (LG3) fragment of endorepellin – the C-terminal domain of perlecan – is produced *via* proteolysis in apoptotic endothelial cells (78). Extracellular matrix remodeling *via* proteolysis, which is associated with the release of cryptic extracellular fragments, is a consequence of membrane degradation (79). LG3 acts as a neoantigen, promoting the production of anti-LG3 antibodies (13, 14). In kidney transplantation, patients with acute vascular rejection were found to have elevated anti-LG3 titers pre- and posttransplantation compared to patients with acute tubulointerstitial rejection or stable graft function. Elevated pretransplant anti-LG3 antibody and pretransplant HLA-DSA levels were both independently associated with acute vascular rejection (13). Of note, anti-LG3 antibodies, which are preformed and persist after transplantation, were associated with chronic lung allograft dysfunction in lung transplant recipients (80). Even if the role of LG3-specific antibodies is increasingly associated with graft failure, the exact underlying mechanisms are still largely unknown. Larger prospective cohort studies need to be performed to better understand the role of these autoantibodies.

### Other Potential Non-HLA Candidates

In addition to the non-HLA antigens cited above, we explored the expression of several other non-HLA antigens suspected or known to be targeted by antibodies linked to allograft rejection, allowing us to study the gene expression of 33 antigens. Among these antigens, *AGRN* (81), *AGT* (82), *PECR* (83), *LIMS1* (84), *ENG* (85), *PPIA* (86), *VIM* (87), *CENPB*, *TRIM21* and *SSB* (88) may have roles in kidney transplant rejection. Interestingly, *CENPB* is broadly expressed in renal cells but not in immune cells. In addition, *VIM*, *ENG, TRIM21* and *PPIA* are highly expressed in activated kidney endothelium, whereas *AGT*, *AGRN*, *PECR*, *LIMS1* and *SSB* seem to be more highly expressed in the epithelium (**Figure 2C**). *HNRNPK* (89), *LMNA* (90) and *ENO1* (91) encode antigens targeted by antibodies in heart rejection. They are expressed in the epithelium and activated endothelial cells. Pathogenic anti-*TUBA1B* antibodies (92) were also described in lung rejection. In the kidneys, this antigen is mainly found in lymphoid cells, but limited expression in endothelial cells has also been detected. Regarding *PTPRN* (93), which is involved in pancreas rejection, and *AURKA* (94), which is involved in skin graft rejection, their expression in the kidneys is weak and mainly occurs in immune cells.

Some of these antigens, such as *FLRT2* (95), *IFIH1* (96) and *GDNF* (87), have been implicated in other diseases and both are highly expressed in the endothelium and vSMp in the kidneys (**Figure 2**), but the link with transplantation needs to be confirmed. Autoantigens corresponding to collagen I to V can also influence the outcome of kidney and lung transplantation (97–99), and their expression is high in cortical endothelial cells (**Figure 2C**).

### Development of New Assays to Identify Non-HLA Antibodies

In recent decades, many antigens have been identified as potential mediators of allograft rejection not only in kidney transplantation but also in heart, lung and liver transplantation. We recommend the review by Kardol-Hoefnagel and Otten, which provides an overview of non-HLA antibodies and their clinical impact on solid organ transplantation (61). What we should remember is that non-HLA antibodies are organ dependent and that each kind of antibody may have a different mechanism of action. To create new solutions for detecting and evaluating the relevance of non-HLA antibodies, a multiplex assay for the detection of antibodies against 14 non-HLA antigens using single-antigen beads evaluated on a Luminex scanner was developed (100). Senev et al. used this assay to investigate 13 of 14 pretransplant non-HLA antibodies and their associations with ABMRh and kidney allograft failure. Of the selected non-HLA antibodies, only the antibodies against ARHGDIB were associated with graft failure, and they synergized with HLA-DSAs (101).

Through a nationwide survey, we identified a highly selected cohort of 38 kidney transplant recipients with homogeneous clinical and pathological presentation of early acute rejection with MVI but no HLA-DSAs. A cellular test was developed to assess non-HLA anti-endothelial cell antibodies. To this end, we developed an *in vitro* assay in which the seroreactivity of serum samples collected on Day 0 was analyzed using the CiGEnC human microvascular glomerular endothelial cell line as the target cells. As CiGEnC cells express class I and class II HLA antigens, this analysis was restricted to ABMRh patients, stable kidney transplant recipients and healthy volunteers with no circulating anti-HLA antibodies to avoid any HLA-dependent cell reactivity. The seroreactivity against CiGEnC cells was significantly increased with ABMRh serum, whereas limited reactivity was observed with serum from healthy volunteers or stable kidney transplant recipients (8). We subsequently applied a CRISPR/Cas9 strategy to delete both the *B2M* and *CIITA* genes by a nonhomologous end-joining pathway to obtain CiGEnCΔHLA cells that can be used to specifically detect non-HLA antibodies even in patients with circulating anti-HLA antibodies. CiGEnCΔHLA cells allowed us to develop a non-HLA antibody detection immunoassay (NHADIA). The evaluation of the NHADIA in an unselected cohort of 389 kidney transplant recipients revealed that preformed non-HLA antibodies were increased in the patients who had undergone previous kidney transplantation, supporting the role of allosensitization to minor histocompatibility antigens. The pretransplant NHADIA value correlated with MVI lesions on the kidney graft at 3 months and 12 months and was associated with the risk of developing ABMRh (102).

### Genome-Wide Analysis to Refine Non-HLA Antibody Identification

Over the last decade, several groups have used a genome-wide approach to identify relevant non-HLA mismatches with a significant association with graft outcome. Mesnard et al. were the first to quantify genome-wide mismatches outside the HLA region. They performed exome sequencing of DNA from 53 kidney transplant recipients and their living donors and estimated all possible cell-surface antigen mismatches for a given donor/recipient pair by computing the number of amino acid mismatches in transmembrane proteins (103). They found a significant effect on the estimated glomerular filtration rate (eGFR) that was independent of HLA-A, HLA-B, DR matching, donor age, and time posttransplantation. Subsequently, Pineda et al. performed whole-exome sequencing with 28 kidney transplant donor/recipient pairs, among whom half developed ABMR. They found a significantly higher number of mismatched non-HLA variants in the pairs with ABMR and identified a set of 123 variants associated with the risk of ABMR (104). More recently, Reindl-Schwaighofer et al. genotyped 477 pairs of deceased donors and first kidney transplant recipients with stable graft function at three months. They analyzed 59268 nonsynonymous single-nucleotide polymorphisms (nsSNPs) affecting a transmembrane or secreted protein, avoiding the MHC locus, and found a median of 1892 mismatches between donors and recipients (105). The degree of nsSNP mismatch was independently associated with graft loss in a multivariable model adjusted for HLA serotype and eplet mismatch. Reindl-Schwaighofer et al. also designed customized peptide arrays that contained both self and non-self peptides (666 peptides) for analysis of 25 patients with a biopsy showing chronic ABMR. They were able to highlight 16 non-HLA antibodies targeting genetically predicted nsSNPs in membrane-associated proteins.

In the Go-CAR patient cohort, Zhang et al. analyzed genome-wide array data, excluding the HLA region, for 385 donor/recipient pairs to study the roles of donor/recipient differences in serial histology and allograft survival. They estimated the ancestry and proportion of genome-shared identity-by-descent (pIBD) in each donor/recipient pair. In donor/recipient pairs with similar ancestry, pIBD was significantly associated with allograft survival independent of HLA mismatching in 224 transplants. Moreover, pIBD was inversely correlated with early vascular intima fibrosis and allograft survival (106). Although rich in valuable information, none of these studies were focused on ABMRh.

### Kidney Endothelium Heterogeneity: The Answer to the Diversity Among Non-HLA Profiles?

Another challenge for non-HLA antibody identification and evaluation of functional consequences is that the phenotype of endothelial cells appears to be extremely heterogeneous depending on the organ and even within individual organs. Chi et al. showed that endothelial cells from different blood vessels or anatomical sites had distinct expression patterns that could play a role in the local physiology or in response to pathology (107). Single-cell RNA sequencing (scRNA-seq) of >40,000 mouse renal endothelial cells highlighted the extensive heterogeneity of these cells, with the identification of 24 (including eight novel) renal endothelial cell phenotypes between and within the cortex, glomeruli, and medulla (108). Knowing that the kidney allograft endothelium is prone to exposure to various injurious stimuli and ischemia–reperfusion injury that disturb the homeostatic function of the endothelium, we can assume that the development of standardized assays to identify non-HLA antibodies applicable in all circumstances is highly challenging. For example, in the study by Delville et al., the IgG reactivity of serum samples from kidney transplant recipients with ABMRh was restricted to glomerular endothelial cells, with no reactivity to primary cultures of human macrovascular endothelial cells or human renal epithelial cells used as targets (8).

The importance of kidney endothelium heterogeneity with respect to the risk of developing non-HLA antibodies was investigated by Li et al., who conducted an integrative genomic analysis of serological responses to non-HLA targets after renal transplantation. They measured posttransplant antibody responses with serum samples from 18 pediatric kidney transplant recipients. Non-HLA responses, including anti-MICA antibodies, were detected against kidney compartment-specific antigens, with the highest posttransplant recognition for renal pelvis- and cortex-specific antigens (109). This study provides an anatomic roadmap of the most likely non-HLA antigens that can generate serological responses after kidney transplantation. However, the correlation with the risk of kidney allograft rejection has to be studied.

All the available data suggest that non-HLA antibodies may play a major role in kidney allograft rejection, as well as in solid organ transplantation in general. Even if their presence is now undeniable, the real challenge is to know whether these antibodies play a role on their own, alone or in a synergistic way with HLA-DSAs, or constitute an epiphenomenon with no pathological effect *per se* (110). In addition, the frequency of non-HLA antibodies in healthy population is not well documented, and a proportion of healthy individuals could have a significant proportion of such antibodies. As an example, Hönger et al. have shown positive levels of anti-AT1R (>17 U/ml) and anti-LG3 (optical density ratio >1) in 19% and 58% healthy controls respectively. Moreover, they found higher anti-AT1R antibodies levels in male controls, compared to women controls (111). As discussed above, these results bring up the question of defining a universal and clinically relevant cut-off for non-HLA antibodies, allowing the discrimination of pathogenic antibodies, possibly depending on gender, age, or other unidentified determinants. Finally, it is still unclear whether a single non-HLA antibody can have a significant impact on graft outcome or whether a combination of several non-HLA antibodies is needed, perhaps with a set of specific antibodies for each individual, therefore requiring potential full sequencing of every donor/recipient pair (105). One approach may be to develop assays allowing us to detect the burden of non-HLA antibody binding to target cells, as we did with our NHADIA (102).

## Mechanisms Involved in Antibody-Mediated MVI

As mentioned above, HLA-DSAs can cause microvascular damage through activation of endothelial cells in the graft, and these cells are directly exposed to the recipient’s immune system. This injury involves activation of the complement system (112) and recruitment of inflammatory cells such as NK cells *via* their crystallizable fragment (Fc) receptors, inducing ADCC (113).

### Complement-Dependent Cytotoxicity and ABMRh

It is now widely accepted that the binding of HLA-DSAs to the graft vasculature, depending on the titer and the heavy chain isotype of the pathogenic antibody, can trigger the classical complement pathway (114, 115). As a marker of local classical complement pathway activation, positive C4d staining in the peritubular capillaries during ABMR has constituted a major criterion of ABMR in previous Banff classifications and is now considered a surrogate criterion for HLA-DSAs (116) when circulating antibodies cannot be identified.

The complement system includes more than 40 proteins, including membrane-bound and soluble proteins, which can act as receptors and regulatory proteins (117). The complement system is activated in the allograft through three distinct pathways: the classical pathway, the lectin pathway, and the alternative pathway. Classical pathway activation is initiated when plasma C1q binds to the Fc segments of IgG and IgM (also called HLA-DSAs). Although these three pathways are engaged by distinct molecular mechanisms, all of them lead to the cleavage of the C3 protein, generating the C3a and C3b fragments (118). Two recent studies have demonstrated that in a nonhuman primate model, C3 complement inhibition can prevent antibody-mediated injury and improve transplant outcomes (119, 120). Ultimately, full complement cascade activation leads to the cleavage of C5, generating the soluble C5a and C5b fragments. Finally, C5b-9 (or the membrane attack complex) forms pores through the outer membrane of endothelial cells and can cause cellular activation, signaling and cell lysis (118).

The presence of C4d in the peritubular capillaries reflects the activation of complement following an antibody-endothelium interaction (**Figure 1**). This link between complement and rejection was confirmed by Feucht et al., who identified the complement cleavage product C4d in transplant biopsies, involving the classical complement pathway in acute and chronic rejection (121, 122). However, several lines of evidence suggest the involvement of complement-independent mechanisms in these types of lesions (32). Although positive C4d staining strengthens the likelihood of a pathogenic role for DSAs, C4d can also be found in the absence of detectable DSAs (123). Conversely, true C4d-negative ABMR can occur in the context of HLA-DSAs (7, 123, 124). An elegant study by Koenig and colleagues compared graft survival among four groups based on the presence of MVI, DSAs and C3d. Interestingly, they found equivalent graft survival between the MVI+DSA+C3d- and MVI+DSA- groups. Moreover, the graft survival of these two groups was better than that of the MVI+DSA+C3d+ group but significantly worse than that of the MVI-DSA- group, suggesting a deleterious impact of MVI on graft survival (7). Finally, in HLA-DSA-negative ABMRh, C4d deposition is also often absent, suggesting once again that complement-independent mechanisms of injury may be involved (56).

### ADCC and ABMRh

Injury to the graft endothelium can also be induced by another mechanism, ADCC. ADCC is initiated by the binding of an antibody (including an HLA-DSA) to its antigenic target by its Fab fragment, which allows the recruitment of effector cells expressing FcγR and having cytolytic potential, such as NK cells, monocytes, macrophages and neutrophils (125) (**Figure 1**). FcγRs are divided into three classes, FcγRI, FcγRII and FcγRIII, corresponding to CD64, CD32 and CD16, respectively, and different forms of the receptors FcγRI, FcγRII (FcγRIIA, FcγRIIB and FcγRIIC), and FcγRIII (FcγRIIIA and FcγRIIIB) have been described (126). Flow cytometry and surface plasmon resonance studies have demonstrated the differences in affinity and avidity among the 3 types of FcγRs with respect to IgG subclasses, with FcγRI presenting the strongest affinity for IgG (127, 128).

CD32 receptors preferentially bind IgG1 and IgG3 and exhibit lower affinity for IgG2 and IgG4 (129). CD32A, CD32B and CD32C are encoded by three genes located at 1q23: FCGR2A, FCGR2B and FCGR2C, respectively. The *CD32A* gene is known to contain a functional polymorphism that affects receptor affinity and impacts innate immune cell functions, such as cytokine production (130, 131). Interestingly, the impact of the *FCGR2A* polymorphism in kidney transplantation is still unclear: although several studies failed to associate it with chronic rejection (132, 133), Yuan et al. showed that the *FCGR2A* polymorphism might be a risk factor for acute kidney graft failure (134). CD32B is highly expressed on the surface of B cells, basophils, approximately 20% of monocytes and 4% of neutrophils but also in a very small fraction of NK cells (135, 136). A SNP has been identified in the *FCGR2B* gene and is associated with receptor dysfunction (137). Of note, in two cohorts including Caucasian and African kidney transplant recipients, Clatworthy et al. found no effects of the *FCGR2B* polymorphism on acute rejection rates, graft function at 1 year, or 10-year transplant or patient survival (138). Structurally, the extracellular and transmembrane domains of CD32C are identical to those of CD32B. CD32C therefore shares the binding specificity of CD32B and induces an activating signal through the signaling pathway of a receptor with an ITAM-like motif identical to that of CD32A (139, 140). CD32C is able to bind complexed human IgG1, IgG3 and IgG4 (127). It is expressed on the surface of NK cells, monocytes/macrophages and neutrophils (126). This receptor has a polymorphism that creates a stop codon, explaining its absence on the surface of NK cells in 50 to 60% of donors (141, 142).

The FcγR type 3 receptor (CD16) exists in two isoforms in humans: FcγRIIIA (CD16A) and FcγRIIIB (CD16B). CD16A and CD16B are encoded by two very homologous genes, and the extracellular region sequences are 96% identical (143). FCGR3A expression is reported in monocytes, NK cells, and a fraction of αβ (144) and γδ T cells (145), as well as in macrophages and certain dendritic cells. In contrast, CD16B expression is limited to polynuclear neutrophils and basophils (146). The *FCGR3A* gene exhibits two polymorphisms that modulate the affinity for IgG (147–149). The relevance of one of these polymorphisms in chronic ABMR was assessed. Eighty-five HLA-DSA+ kidney allograft recipients who were recruited upon antibody screening of 741 prevalent patients were genotyped, and the patients with high-affinity polymorphic CD16A showed a higher rate of ptc in protocol biopsies. In parallel, there was a trend toward increased myeloid-associated transcripts in these patients (132). Interestingly, a very recent study using scRNA-seq analysis of kidney transplant patients reported that FCGR3A was highly expressed by graft-infiltrating monocytes during ABMR (150), suggesting that these monocytes can play a pathogenic role during rejection by infiltrating the graft and performing ADCC subsequent to DSA fixation.

Altogether, these data suggest an important role for immune cells armed with Fc receptors. Despite the important findings for FcR polymorphisms and their clear involvement in solid organ rejection, no assessment has yet been translated into clinical diagnostic parameters. Moreover, it is unknown whether non-HLA antibodies can trigger microvascular damage through a mechanism such as ADCC, regardless of whether FcR polymorphisms are involved. Wide mechanistic studies are needed to understand how non-HLA antibodies can exert a deleterious effect on the graft endothelium.

## Heterogeneity of Infiltrating Immune Cells During ABMRh

**Figure 3:**
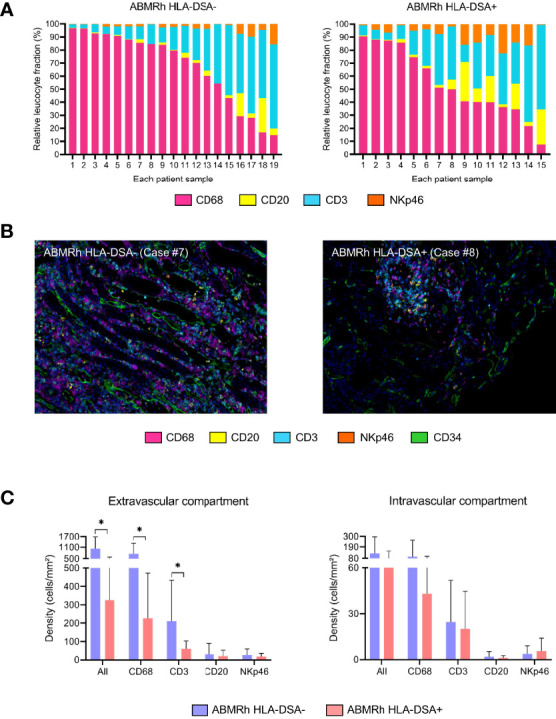
Important heterogeneity of infiltrated immune cells in kidney allografts with microvascular inflammation. **(A)** Relative fractions of CD68+ cells, CD3+ cells, CD20+ cells, and NKp46+ cells for each case of antibody-mediated rejection without (left) or with (right) circulating anti-HLA donor-specific antibodies (HLA-DSAs). **(B)** Representative image of multiplex immunofluorescence staining showing macrophages (purple), T cells (blue), B cells (yellow), and NK cells (orange) in a case of HLA-DSA- ABMR (left; Case #7) or HLA-DSA+ ABMR (right; Case #8). Endothelial cells are shown in green (CD34+) and identify peritubular capillaries. **(C)** Cellular densities according to the detection of circulating HLA-DSAs in the extra- and intravascular compartments. HLA-DSA-negative ABMR samples display more inflammatory cells (CD68+ and CD3+) in the extravascular compartment. Leukocyte densities are expressed as the number of cells per square millimeter (mm2), and relative fractions are expressed as percentages (%). Statistical analyses were performed with Prism software version 9.3.1 (GraphPad Software). We compared the means of 2 groups using the Mann–Whitney *U* test. Values are given as the mean ± SD. *P < .05.

Little is known about the exact composition of immune cell infiltrates in the kidney allograft in patients with TCMR or ABMR. In a recent study from our laboratory (151), T lymphocytes and macrophages represented the 2 major populations during rejection, either TCMR or ABMR, whereas NK cells represented a very small proportion of the total inflammatory burden. Even if the proportions of NK cells and macrophages are quite similar between TCMR and ABMR, these populations of cells appear to be compartment specific within the nephron. Indeed, macrophage and NK cell counts were found to be significantly higher in the peritubular capillaries during ABMR than during TCMR (151, 152). However, no study has compared the composition of the infiltrates during ABMR with HLA-DSAs to that during ABMR without HLA-DSAs. Interestingly, in our study, in a small cohort of 20 patients with ABMR, including 4 lacking HLA-DSAs, the frequencies of NK cells, macrophages and T cells were very similar between patients with ABMR with or without HLA-DSAs (151). To provide a larger-scale study, we assessed the proportions of macrophages, B cells, T cells, and NK cells using multiplex immunofluorescence as previously published (151) with automated quantification of CD68-, CD20-, CD3- and NKp46-positive cells in a cohort of 15 HLA-DSA+ ABMR and 19 HLA-DSA- ABMR patients (**Figure 3**). We found important heterogeneity in the inflammatory burden during kidney allograft rejection, in either the presence or absence of HLA-DSAs, and very similar proportions of macrophages, B cells, T cells, and NK cells were found in the biopsies with or without circulating HLA-DSAs (**Figures 3A, B** **)**. NK cells and B cells were the least represented cell types among all cell populations but still exhibited high variability across the biopsies. The extravascular density was higher in the HLA-DSA- ABMR group, with higher densities of macrophages and T cells, than in the HLA-DSA+ ABMR group (**Figure 3C**), which was also correlated with higher total inflammation and tubulitis scores according to the Banff classification.

These findings confirm that the composition of the inflammatory burden during ABMR, with or without circulating HLA-DSAs, is much more complex than previously thought, exhibiting high variability among individuals, and may not be useful for characterizing the mechanisms involved.

## Antibody-Independent MVI

Antibodies directed against donor-derived antigens cause inflammation in the microvascular endothelium, an undeniable marker of ABMR. MVI is characterized by local activation of the complement system and accumulation of innate and adaptive immune cells within the capillaries. A traditional view in transplantation is that adaptive immune cells (i.e., T and B lymphocytes) and specialized surface-expressed proteins encoded by genes within the MHC complex (also known as the HLA complex) are necessary and sufficient for allograft rejection. However, the vascular injury in an allograft in the effector phase of rejection is probably far more complex and could involve many other cell types, as suggested by our results (**Figure 3**), thereby complicating the current view of allograft rejection. Unlike the knowledge on B and T cells, little is known about how innate immune cells participate in the allograft response. Perhaps slightly naively, transplant physicians have long considered that most innate cells do not respond directly to allotransplants but are instead recruited by activated T cells during the process of graft destruction. However, several teams have recently reported evidence of a direct role for innate cells in allograft rejection. NK cells and monocytes/macrophages are able to distinguish between self and non-self and trigger an alloimmune response (153). Recruitment of these cells in response to nonspecific “danger” signals from various causes (for example, ischemia–reperfusion after organ transplantation) could elicit inflammation associated with tissue injury.

### NK Cell-Mediated Rejection

The current doctrine is that MVI triggered by an antibody response against an alloantigen is the main cause of graft failure. Among innate immune cells, populations expressing the Fc receptor family (CD16, CD32 or CD64), which makes them able to recognize any antibody, are considered potential effectors in ABMR. NK cells are cytotoxic lymphocytes expressing the surface markers CD56 and CD16. Depending on their expression patterns, these cells can have strong cytotoxic (CD56^dim^ CD16^bright^) or regulatory (CD56^bright^ CD16^dim^) properties. In the indirect recognition mechanism called ADCC, the expression of CD16 by NK cells is increased, which enables antibody-coated target cell detection (154). Sablik et al. reported an increase in CD16 expression on circulating NK cells in recipients with active chronic ABMR compared to matched recipients without ABMR (155). In line with these findings, certain groups reported that the density of NK cells was significantly higher in patients presenting ABMR than in patients without rejection or with TCMR (151, 156).

The activity of NK cells is controlled by the complex interplay between activating and inhibitory receptors that mainly belong to either the C-type lectin-like receptor (CLR) superfamily or the killer immunoglobulin receptor (KIR) family. KIR receptors recognize HLA class I molecules and can be either activating or inhibitory. Inhibitory KIRs play an important role in the maturation of NK cells by mediating their “education”, making the cells ready to attack cells not expressing the corresponding ligand, a mechanism called “missing self”. This education is necessary to prevent NK cells from becoming autoreactive and depleting their cytotoxic granules, as is the case when malignant or virally infected cells downregulate surface HLA class I expression. In the case of transplantation, if donor endothelial cells express an HLA I allotype that is unable to interact with a KIR receptor expressed by the recipient’s NK cells, these recipient NK cells would be likely to attack through the missing self mechanism (157) (**Figure 1**).

It is well established that NK cells participate in the microvascular inflammatory response in HLA-DSA-positive ABMR (156, 158). The question is whether NK cells can mediate MVI independent of any antibodies. Olivier Thaunat’s group showed that NK cells could induce histological lesions independent of antibody mediation (7). In a cohort of 129 kidney recipients with MVI on graft biopsy, no antibodies were detected in approximately half of the participants. Genetic analysis of the patients showed that 65.1% presented a genetically predicted mismatch between donor class I HLA and recipient inhibitory killer cell immunoglobulin-like (iKIR) receptors. Using both *in vitro* models and transplantation of *β2-microglobulin-*deficient hearts into wild-type mice, they demonstrated that NK cells could allorecognize donor endothelial cells in the case of recipient KIR/donor HLA mismatch and thus participate in endothelial injury. Subsequently, the same group analyzed 135 HLA-DSA+MVI+ ABMR kidney transplant recipients. The 73 patients with complement-fixing DSAs identified by a positive C3d binding assay result had a higher risk of transplant failure. Within the C3d- group, the patients in whom missing self was identified through donor/recipient genotyping exhibited worse allograft survival. Furthermore, cocultures of human NK cells and endothelial cells confirmed that missing self synergized with DSAs to increase endothelial damage (159).

Despite all these emerging data, we can assume that NK cells are not the only innate immune effectors involved in MVI lesions, since some patients with MVI on graft biopsy but no antibodies do not have a genetically predicted missing self mismatch (7). Interestingly, the involvement of NK cells during allograft rejection was first suggested by transcriptomic analyses. Comparison of biopsies from DSA-negative *versus* DSA-positive patients highlighted 132 differentially expressed transcripts. Among them, 6 transcripts linked to NK cells were found to have selectively higher expression (158). Very recently, Callemeyn et al. confirmed these findings by analyzing transcriptional changes in 56 biopsies from patients with ABMRh. They found overexpression of transcripts mostly related to IFNγ-induced pathways and NK cell activation (9). However, IFN-γ signaling pathways are involved in the transcriptional regulation and activation of many immune cells, including NK cells, T cells, macrophages and dendritic cells (160). Therefore, the IFNγ-induced transcripts that are considered markers of NK cell involvement may also be expressed by other cell types (e.g., monocytes and T cells). Moreover, it has been shown in a murine allograft model that host macrophage depletion but not T cell, NK cell, neutrophil, or complement depletion inhibits *in vivo* allocytotoxicity. Finally, as shown by our results, macrophages are the most represented population within the graft during ABMR (**Figure 3**). Together, these studies also reveal the potential role of macrophages as effectors in endothelial injury during ABMR.

### Emerging Data Reveal the Role of Monocytes/Macrophages in Kidney Transplantation

The role of recipient monocyte-derived macrophages following ischemia–reperfusion injury and vascular anastomosis is now well established (161, 162). Moreover, it has been shown that activated macrophage infiltrates are present during clinical rejection and could be responsible for acute graft dysfunction (163). Monocytes circulate in the blood, bone marrow, and spleen, composing between 2% and 10% of all leukocytes in the human body. Their main role under normal conditions is to replenish resident macrophages in tissues, but they can differentiate into inflammatory dendritic cells or macrophages during inflammation and migrate into tissues in response to “danger” signals (164). In a target tissue, monocytes and their macrophage progeny serve four main functions in the immune system. These are phagocytosis, antigen presentation, cytokine production and killing of infected host cells *via* ADCC. In transplantation, *in vitro* and *in vivo* experiments have shown that alloantibody-dependent allograft rejection is mediated by host macrophages through FcγRs and reactive oxygen species (ROS)-related cytotoxic effector mechanisms (165). In biopsies of kidney allografts, CD68 and CD163 are two main markers used to follow the presence of tissue-resident macrophages and monocyte-derived macrophages (151, 152, 166). The density of CD68^+^ macrophages in early surveillance kidney allograft biopsies has been shown to be a significant predictor of allograft dysfunction four years after transplantation (167). Increased CD68^+^ macrophage infiltration was shown to be significantly elevated in both ABMR and TCMR but not in kidneys with established interstitial fibrosis and tubular atrophy. Biopsies with ABMR showed mainly peritubular infiltration, whereas those with TCMR were correlated with CD68^+^ cells in the peritubular and perivascular compartments, suggesting compartment-specific infiltration and proliferation of these cells (152). We also found a significant and equivalent proportion of CD163^+^ macrophages in both ABMR and TCMR biopsies compared to normal biopsies. However, the macrophage density in the glomerular and peritubular capillaries was significantly higher during ABMR (151). In a cohort of 52 ABMR cases, the number of CD68+ macrophages in the capillaries and interstitium was correlated with the HLA-DSA MFI and associated with allograft loss at the time of ABMR diagnosis (168).

In murine transplant models, macrophages were shown to be recruited by non-self determinants and to act as direct effectors of rejection (169). Moreover, monocyte depletion was shown to prevent loss of the renal microvasculature in a murine model of acute kidney rejection (170). In a murine heterotopic cardiac transplant system known to develop cardiac allograft vasculopathy, macrophage depletion led to a 70% reduction in lesions compared to mock treatment (171). In terms of mechanism, monocytes were shown to be able to distinguish self and non-self allogeneic antigens independently of NK and T cell-dependent alloresponses (172, 173). More importantly, recent data from Lakkis’s group highlighted this allorecognition driven by monocytes. Notably, in a murine *Rag^2-/-^γc^-/-^* model, which lacks T, B and NK cells, the activation of monocytes infiltrating the graft was mediated by binding of donor signal regulatory protein alpha (SIRPα) to recipient CD47 in a manner independent of T, B, and NK cells (**Figure 1**). By phylogenetic analysis, they revealed that donor SIRPα polymorphism controlled the host innate alloresponse by modulating the binding of SIRPα to CD47. Allelic variation in donor SIRPα could cause greater CD47/SIRPα binding, resulting in CD47 signaling and increased monocyte activation and dendritic cell transformation. This binding strength makes monocytes able to distinguish between self and allogeneic non-self (174). Using a similar murine model established with *Rag^2-/-^Il2rg^-/-^* mice, which lack B, T, NK and innate lymphoid cells, the same team later showed that monocytes and macrophages acquired memory specific to MHC-I antigens mediated by paired Ig-like receptors (PIRs). Indeed, deletion of PIR-A in the recipient or blocking of PIR-A binding blocked memory and decreased the risks of kidney and heart rejection (175). Altogether, these recent reports suggest a central role for monocyte/macrophage allorecognition in graft rejection and monocyte-driven rejection (**Figure 1**).

## Conclusion

**Figure 4:**
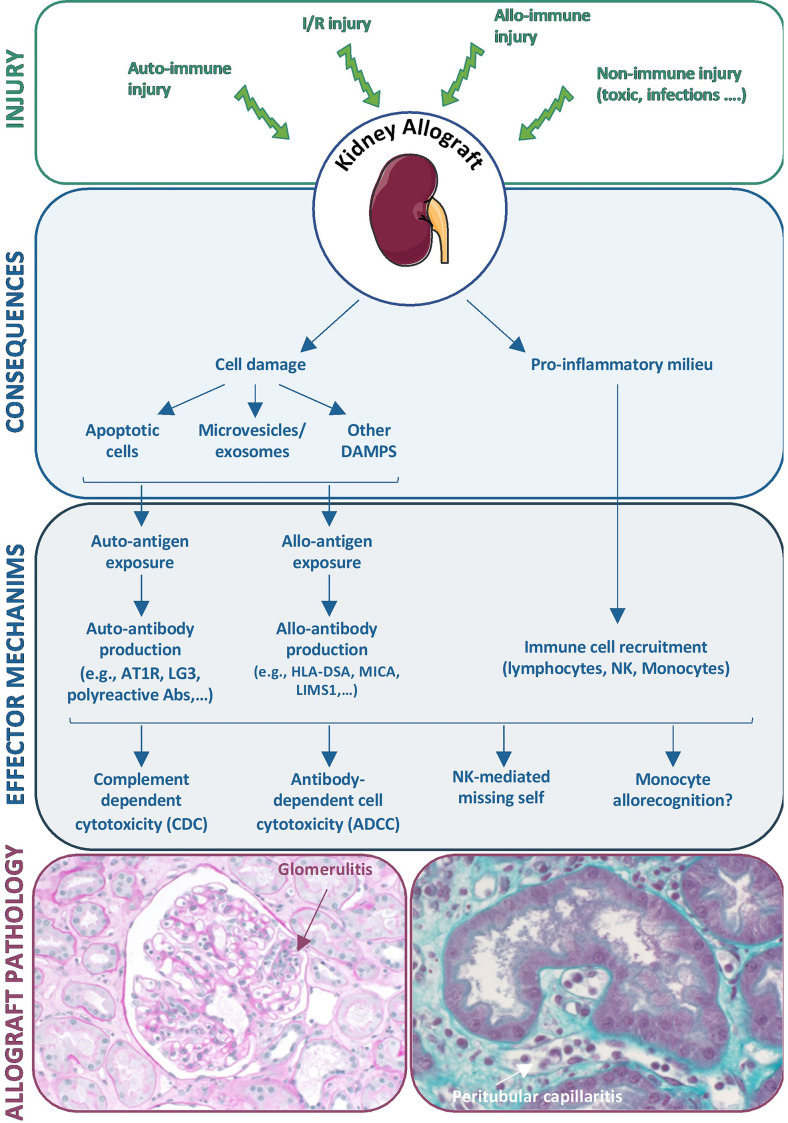
Diversity of mechanisms underlying microvascular inflammation in the kidney allograft. Diverse injuries to the kidney allograft can induce cell damage and a pro-inflammatory environment that initiate diverse effector mechanisms including antibody production and recruitment of immune cells, both leading to allograft lesions of ABMRh.

Recent experimental and clinical research highlights the wide diversity of mechanisms underlying MVI in the kidney allograft, the prototypic histological lesion of the immune injury called ABMR. Injury to graft endothelial cells can of course be triggered by antibodies recognizing non-self HLA molecules, but a wide diversity of non-HLA auto- and alloantibodies can also be involved. In addition, nonantibody-mediated mechanisms of graft endothelial cell injury, including NK cell-mediated missing self and allorecognition by host monocytes, are being identified (**Figure 4**). This complex interplay between antibody-dependent and antibody-independent injuries and the involvement of many effector cells exemplifies that a single effector cannot induce a complete phenotype by itself and that the immune response is far more complex than expected. Thus, the ABMR spectrum should be viewed as the integration of many synergistic mechanisms that can play roles in the allograft rejection process. We believe that this underrecognized diversity among pathogenic mechanisms largely explains the relatively disappointing results for ABMR treatment in clinical practice. Should we classify and treat ABMR according to the major immunological process involved in the observed rejection? Whether such an approach is feasible, it would require the development of reliable and specific biomarkers to guide highly tailored therapeutic strategies.

## Author Contributions

EL, BL, and DA wrote the manuscript. ME and MR have conducted the study for the **Figure 3**. All authors have reviewed and approved the final version of the manuscript. BL and DA have contributed equally to this work and share last authorship.

## Funding

This work was supported by the Fondation du Rein sous l’égide de la Fondation pour la Recherche Médicale (Prix Don de Soi-Don de Vie 2018 FdR/FRM_D.ANGLICH Subvention Transplantation et Thérapie Cellulaire 2020 FRM PME20200611626; to DA), by the Day Solvay Foundation and by the Boussard Foundation.

## Conflict of Interest

The authors declare that the research was conducted in the absence of any commercial or financial relationships that could be construed as a potential conflict of interest.

## Publisher’s Note

All claims expressed in this article are solely those of the authors and do not necessarily represent those of their affiliated organizations, or those of the publisher, the editors and the reviewers. Any product that may be evaluated in this article, or claim that may be made by its manufacturer, is not guaranteed or endorsed by the publisher.
